# The Profession and the Ministry

**Published:** 1921-05-28

**Authors:** 


### THE PROFESSION AND THE MINISTRY.

In view of the various and often unreasonable attacks which have, from its early days, been made upon the Ministry of Health, it is interesting to note the reception given by the professional Press to Sir Alfred Mond's statement in respect of the present position and future intentions of his department. Just as his explanation resulted in an agreement to the expenses vote without any damaging criticism, so the general public should now have a far clearer conception of the economy of spending money in the prevention of disease.

One is glad to see that both the leading medical journals have drawn particular attention to the fact that charges of extravagance in the appointment of medical officers under the Ministry have completely broken down. During the debate a good deal of the short time available was occupied in getting at the truth of this really simple fact, and the *Lancet* has now carefully summarised the statements of both Sir Alfred Mond and his predecessor. They are in complete agreement. Omitting the regional referees of the Insurance scheme, whose appointment was indeed approved by Parliament in 1914 but suspended during the war, there is a net increase of only thirteen medical officers. In any case it was obvious, in view of sanitary control necessary in any post-war period, that an adequate medical staff was necessary and some increase in numbers therefore inevitable.

Some progress in the housing schemes is now claimed, but there is also general approval of all attempts at investigating and cutting down estimates in outlying districts and concentrating upon the great industrial centres in which the housing need is greatest. Sir Alfred Mond gives 300,000 houses as a gross requirement figure, but is apparently prepared to reduce it still further if he can find justification for doing so. Already it is known that some local authorities considerably exaggerated the needs of their districts, and it is still uncertain whether sufficient account has been taken of the possibilities of repairing all the old houses available.

That a new form of State medical service is not in contemplation is expectedly well received, as also the fact that attempts are being made to improve the existing panel system while retaining free choice of doctors. We heartily join with the *Lancet* in the hope that one of these attempts will be official encouragement of the approved societies to assist in the support of the voluntary hospitals. Already these societies are discussing with the British Hospitals Association facts concerning institutional treatment of their members, who form a large proportion of the panel patients. From every point of view the question of really efficient Insurance practice presents great difficulties, but it is probable that the new Minister is right in his opinion that the regional referees, whose duties are largely concerned with the members of the approved societies, will fully justify their appointment. In any case, the medical profession's general reception of Sir Alfred Mond's explanations should be ample illustration to the lay public that whatever Press criticisms have been advanced in the past, the efforts already made by the Ministry have been by no means in vain.

